# Study on Synthesizing Isobornyl Acetate/Isoborneol from Camphene Using α-Hydroxyl Carboxylic Acid Composite Catalyst

**DOI:** 10.3390/molecules28041875

**Published:** 2023-02-16

**Authors:** Zhong-Lei Meng, Rong-Xiu Qin, Ru-Si Wen, Gui-Qing Li, Zhong-Yun Liang, Jun-Kang Xie, Yong-Hong Zhou, Zhang-Qi Yang

**Affiliations:** 1 Guangxi Key Laboratory of Superior Timber Trees Resource Cultivation, Guangxi Forestry Research Institute, Nanning 530002, China; 2 Institute of Chemical Industry of Forest Products, Nanjing 210042, China; 3 College of Chemical Engineering, Forestry University, Nanjing 210037, China

**Keywords:** α-hydroxyl carboxylic acid, camphene esterification reaction, camphene hydration reaction, green synthesis

## Abstract

This study examined the preparation of isobornyl acetate/isoborneol from camphene using an α-hydroxyl carboxylic acid (HCA) composite catalyst. Through the study of the influencing factors, it was found that HCA and boric acid exhibited significant synergistic catalysis. Under optimal conditions, when tartaric acid–boric acid was used as the catalyst, the conversion of camphene and the gas chromatography (GC) content and selectivity of isobornyl acetate were 92.9%, 88.5%, and 95.3%, respectively. With the increase in the ratio of water to acetic acid, the GC content and selectivity of isobornol in the product increased, but the conversion of camphene decreased. The yield of isobornol was increased by adding ethyl acetate or titanium sulfate/zirconium sulfate to form a ternary composite catalyst. When a ternary complex of titanium sulfate, tartaric acid, and boric acid was used as the catalyst, the GC content of isobornol in the product reached 55.6%. Under solvent-free conditions, mandelic acid–boric acid could catalyze the hydration reaction of camphene, the GC content of isoborneol in the product reached 26.1%, and the selectivity of isoborneol was 55.9%. The HCA–boric acid composite catalyst can use aqueous acetic acid as a raw material, which is also beneficial for the reuse of the catalyst.

## 1. Introduction

Isobornyl acetate smells like pine needles, and it is a safe fragrance ingredient whose global usage exceeds 1000 tons/year [1,2]. Another important use of isobornyl acetate is to synthesize isoborneol by saponification. Isoborneol has a camphor-like smell, is an intermediate in camphor synthesis, and is also a safer and more widely used fragrance ingredient [3,4].

Camphene and acetic acid, as raw materials, can be used to synthesize isobornyl acetate through acid catalysis. At first, sulfuric acid was often used as a catalyst, but due to equipment corrosion and environmental issues, its use was stopped [5]. In order to solve the environmental issues, heterogeneous catalysts such as cation exchange resin [6], sulfonated crosslinked polystyrene balls [7], zeolite molecular sieves [8], solid superacid MoO_3_/ZrO_2_ [9], SO_4_^2−^/TiO_2_ supported phosphotungstic acid solid acid catalysts [10], and ionic liquids [11,12] have been used as catalysts, providing good catalytic effects. In industry, a strong acid cation resin is often used as a catalyst, and the coverage of polymers generated by the reaction on the catalyst activity centers can easily decrease the catalytic activity [6,13]. The preparation of heterogeneous catalysts is complex and costly, and thus, cheap Lewis acids have also been studied as catalysts. Among these catalysts, anhydrous FeCl_3_ had the best catalytic effect; one study found that when the amount of catalyst was 10% of the mass of camphene, the conversion of camphene was 98%, the yield of isobornyl acetate was 88%, and the selectivity was 94.2% [14].

Because an important use of isoborneol acetate is the synthesis of isoborneol, the direct hydration of camphene to prepare isoborneol would be beneficial. A strong acid cation exchange resin can catalyze the reaction between camphene and water, and it requires solvents such as isopropanol. The by-products of this reaction are isobornyl isopropyl ether and fenchyl alcohol [15]. Phosphotungstic acid is also used to catalyze the reactions of camphene and acetic acid or water, and its activity is stronger than those of H_2_SO_4_ and Amberlyst-15 [16,17]. The content of isoborneol in the products obtained from the existing catalytic reaction is low, and the reaction requires a large amount of organic solvent [17,18,19]. For the direct hydration reaction of camphene, it is important to improve the yield of isobornol or not use organic solvents.

α-hydroxyl carboxylic acid (HCA) is a fruit acid that is non-toxic and renewable, and it can be used as a catalyst to meet the requirements of green chemistry. HCA has a low catalytic activity for the esterification and hydration of camphene, so it is necessary to improve its catalytic activity. The valence electron structure of the B atom is 2s^2^2p^1^, and the number of valence electrons is less than the number of valence orbitals. However, compared with the metal elements lithium and beryllium of the same period, boron has a smaller atomic radius, higher ionization energy, and greater electronegativity, which is characterized by the formation of covalent bond molecules. With sp^2^ electrons in the covalent molecule formed by hybridization, the remaining empty orbital of the boron atom can be used as a Lewis acid to accept foreign lone pair electrons and form sp^3^ hybrid tetrahedral complexes. When tartaric acid and boric acid form a coordination compound, the conductivity and optical rotation increase [19,20]. The structures, reaction equilibria, reaction kinetics, and thermodynamics of the complexes formed by boric acid and AHAs have been previously reported [20,21]. B, N co-doped carbon surfaces and ultra-small Ru nanoparticles can transfer electrons from N atoms to Ru and from Ru to B atoms in reverse, which provides a moderate electronic modification of Ru, thus improving the catalytic activity [22].

In this paper, the HCA composite catalyst was used to catalyze the synthesis of isobornyl acetate/isobornol from camphene, and the solvent-free synthesis of isobornol was explored. The synergistic catalytic effect of HCA and boric acid was better than that of a single catalyst. Compared with an anhydrous FeCl_3_ catalyst, the composite catalyst had a stronger tolerance to water and could be recovered. The study of the synergistic catalysis of hydroxycarboxylic acid and boric acid may provide a reference for the development and design of heterogeneous catalysts with carboxylic acid groups.

## 2. Results and Discussion

### 2.1. Catalyzed Synthesis of Isobornyl Acetate from Camphene

#### 2.1.1. Effect of α-Hydroxyl Carboxylic Acid (HCA) and Boric Acid Dosage on Catalytic Performance

**Figure 1:**
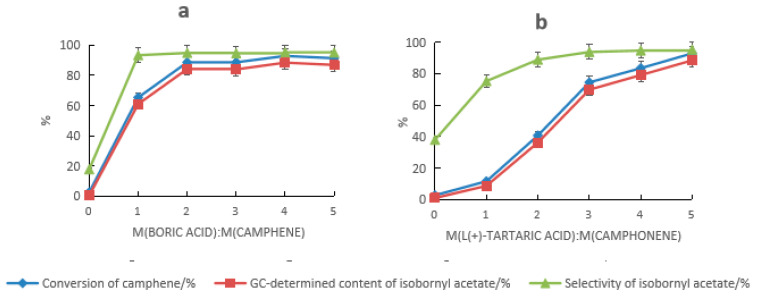
Effect of composition changes of the tartaric acid–boric acid composite catalyst on the catalytic performance. (**a**) Effect of tartaric acid on the catalytic performance under the following reaction conditions: m(camphene):m(acetic acid):m(boric acid) = 10:25:0.4 (where m refers to mass ratio), reaction temperature of 70 °C, and reaction time of 18 h. (**b**) Effect of boric acid on the catalytic performance under the following reaction conditions: m(camphene):m(acetic acid):m(tartaric acid) = 10:25:0.5, reaction temperature of 70 °C, and reaction time of 18 h.

**Figure 2:**
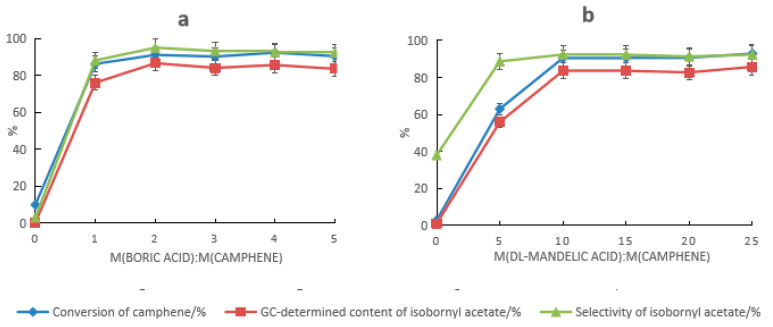
Effect of composition changes in the mandelic acid–boric acid composite catalyst on the catalytic performance. (**a**) Effect of mandelic acid on the catalytic performance under the following reaction conditions: m(camphene):m(acetic acid):m(boric acid) = 10:10:0.5, reaction temperature of 70 °C, and reaction time of 18 h. (**b**) Effect of boric acid on the catalytic performance under the following reaction conditions: m(camphene):m(acetic acid):m(mandelic acid) = 10:25:1, reaction temperature of 70 °C, and reaction time of 18 h.

HCA and boric acid provided a distinct synergistic catalysis effect. When HCA alone was used as the catalyst, the conversion rate of camphene and the selectivity of isobornyl acetate were both low. When tartaric acid was used alone, they were 2.6% and 17.8%, respectively, and when mandelic acid was used alone, they were 10% and 3.1%, respectively. The results are shown in Figure 1a and Figure 2a. When HCA and boric acid constituted the composite catalyst, the conversion rate of camphene and the GC content and selectivity of isobornyl acetate increased with the increase in the boric acid dosage. When the dosage of tartaric acid and boric acid were 5% and 4% of the mass of the camphene, respectively (at this time, the molar ratio of boric acid to tartaric acid was approximately 2:1), the conversion rate of camphene and the GC content and selectivity of isobornyl acetate were the highest, with values of 92.9%, 88.5%, and 95.3%, respectively, as shown in Figure 1a. When the dosages of mandelic acid and boric acid were 10% and 2% of the mass of camphene, respectively, the conversion rate of camphene and the GC content and selectivity of isobornyl acetate were the highest, with values of 91.2%, 86.7%, and 95.1%, respectively, as shown in Figure 2a. This was because boric acid reacted with HCA’s hydroxyl groups, which formed a coordination compound, improving HCA’s ability to donate protons.

When HCA and boric acid formed the composite catalyst, the impact of HCA on the performance of the composite catalyst was greater than that of boric acid, as shown in Figure 1b and Figure 2b. It can be seen from Figure 1b that a sufficient amount of tartaric acid was required to promote the conversion of camphene. When the dosage was ≤4%, the conversion rate of camphene was less than 90%. Similarly, it can be seen from Figure 2b that a sufficient amount of mandelic acid was needed to promote the conversion of camphene. When the dosage was ≥10%, the conversion rate of camphene could reach more than 90%. When the dosage of the catalyst was the same, the catalytic activity of the tartaric acid composite catalyst was higher than that of the mandelic acid composite catalyst. Compared with the anhydrous FeCl_3_ catalyst, the amount of the tartaric acid–boronic acid composite catalyst required was lower, and the yield and selectivity of isobornyl acetate were higher.

#### 2.1.2. Effect of Acetic Acid Dosage on Camphene Esterification Reaction

**Figure 3:**
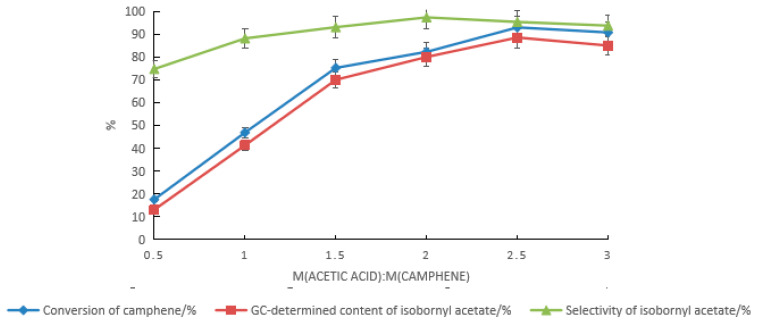
Effects of the acetic acid dosage on the camphene esterification reaction. Reaction conditions: m(camphene):m(tartaric acid):m(boric acid) = 10:0.5:0.4, reaction temperature of 70 °C, and reaction time of 18 h.

Based on the composite catalyst of tartaric acid and boric acid, the effect of the acetic acid dosage on the synthesis of isobornyl acetate from camphene was examined, as shown in Figure 3. With the increase in the acetic acid dosage, the conversion rate of camphene and the GC content and selectivity of isobornyl acetate gradually increased. When the mass ratio of acetic acid to camphene was 2.5:1, the conversion rate of camphene and the GC content and selectivity of isobornyl acetate were the highest, with values of 92.9%, 88.5%, and 95.3%, respectively. When the acetic acid dosage continued to increase, the conversion rate of camphene and the GC content and selectivity of isobornyl acetate were slightly reduced.

#### 2.1.3. Effect of Temperature Changes on Camphene Esterification Reaction

**Figure 4:**
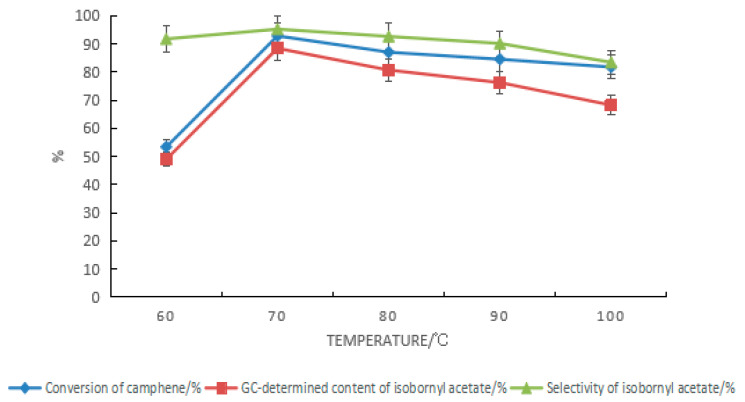
Effect of temperature changes on the camphene esterification reaction. Reaction conditions: m(camphene):m(acetic acid):m(tartaric acid):m(boric acid) = 10:25:0.5:0.4, reaction temperature of 70 °C, and reaction time of 18 h.

**Figure 5:**
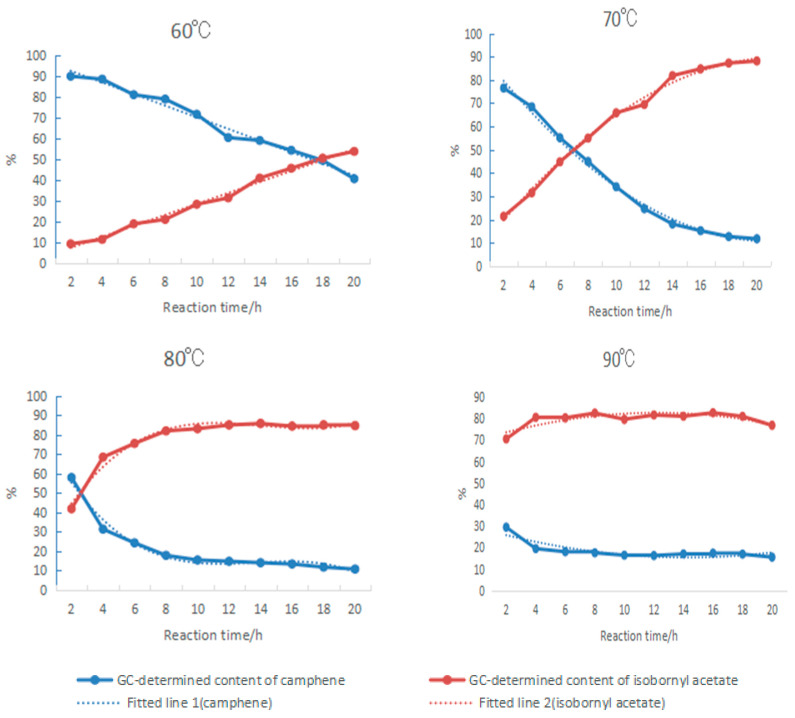
Variations in GC contents of camphene and isobornyl acetate in the product with increasing reaction time. The mass ratio between camphene, acetic acid, tartaric acid, and boric acid was 10:25:0.5:0.4.

For the composite catalyst composed of tartaric acid and boric acid, the impact of the reaction temperature on the synthesis of isobornyl acetate from camphene is shown in Figure 4. The elevated temperature was conducive to speeding up the reaction, but because the reaction was reversible, after the temperature increased, the rate of camphene formation was accelerated by the removal of carboxyl groups from the isobornyl acetate, resulting in reduced conversion of camphene. At the same time, the increase in temperature also led to more side reactions, which caused a decrease in the GC content and selectivity of isobornyl acetate. It can be seen from Figure 5 that the optimal reaction temperature for the synthesis of isobornyl acetate from camphene was 70 °C when the tartaric acid–boric acid composite catalyst was used.

#### 2.1.4. Effect of Changes in Reaction Time on Camphene Esterification

Using tartaric acid and boric acid as composite catalysts, the changes in camphene and isobornyl acetate in the product with increasing reaction time were investigated at 60 °C, 70 °C, 80 °C, and 90 °C, as shown in Figure 5. The mass ratio of acetic acid to camphene was 25:10, which was equivalent to a molar ratio of 5.67:1. Due to the excess amount of acetic acid, it could be assumed that the concentration of acetic acid was constant during the reaction. By fitting the experimental data in Figure 5, the relationships between the contents of camphene and isobornyl acetate and the reaction time were obtained, as shown in the following formulas:

(1) $$y1\;\left(60\;{}^{\circ}C\right)=-2.7397x\;+98.13,\;R^{2}=0.9828,$$

(2) $$Y2\;\left(60\;{}^{\circ}C\right)=2.63695x\;+2.1478,\;R^{2}=0.9896,$$

(3) $$Y1\;\left(70\;{}^{\circ}C\right)=0.186675\times {}^{2}-7.932x\;+94.689,\;R^{2}=0.9943,$$

(4) $$y2\;\left(70\;{}^{\circ}C\right)=-0.17665{x\;}^{2}+7.748x\;+4.992,\;R^{2}=0.9946,$$

(5) $$y1\;\left(80\;{}^{\circ}C\right)=-0.0292125x^{3}+1.197075x^{2}-15.9x\;+82.638,\;R^{2}=0.9772,$$

(6) $$y2\;\left(80\;{}^{\circ}C\right)=0.0271x^{3}-1.149925x^{2}+15.5725x\;+17.952,\;R^{2}=0.9728,$$

(7) $$y1\;\left(90\;{}^{\circ}C\right)=0.068975x^{2}-1.9673x\;+29.448,\;R^{2}=0.7315,$$

(8) $$y2\;\left(90\;{}^{\circ}C\right)=-0.08595x^{2}+2.10025x\;+69.748,\;R^{2}=0.6629,$$

where y1 represents the GC content of camphene in the product (%), y2 represents the GC content of isobornyl acetate (%), x represents the reaction time (h), and *R*^2^ is the determination coefficient.

The derivatives of Formulas (1)–(8) can be used to obtain the change rate of the GC contents of camphene and isobornyl acetate with increasing reaction time:

(9) $$v1\;\left(60\;{}^{\circ}C\right)=\frac{\mathrm{dy}1}{\mathrm{dx}}=-2.7397$$

(10) $$v2\;\left(60\;{}^{\circ}C\right)=\frac{\mathrm{dy}2}{\mathrm{dx}}=2.63695$$

(11) $$v1\;\left(70\;{}^{\circ}C\right)=\frac{\mathrm{dy}1}{\mathrm{dx}}=0.37335x-7.932$$

(12) $$v2\;\left(70\;{}^{\circ}C\right)=\frac{\mathrm{dy}2}{\mathrm{dx}}=-0.3533x\;+7.748$$

(13) $$v1\;\left(80\;{}^{\circ}C\right)=\frac{\mathrm{dy}1}{\mathrm{dx}}=-0.0876375x^{2}+2.39415x-15.9$$

(14) $$v2\;\left(80\;{}^{\circ}C\right)=\frac{\mathrm{dy}2}{\mathrm{dx}}=0.0813x^{2}-2.29985x\;+15.5725$$

(15) $$v1\;\left(90\;{}^{\circ}C\right)=\frac{\mathrm{dy}1}{\mathrm{dx}}=0.13795x-1.9673$$

(16) $$v2\;\left(90\;{}^{\circ}C\right)=\frac{\mathrm{dy}2}{\mathrm{dx}}=-0.1719x\;+2.10025$$

where v1 represents the change rate of the GC content of camphene in the product with increasing reaction time, v2 represents the change rate of the GC content of isobornyl acetate with increasing reaction time, and x represents the reaction time (h).

Formulas (9)–(16) show that when camphene reacted with acetic acid, the reaction rate increased with the increase in temperature. At 60 °C, the reaction rate was constant. When the temperature was above 70 °C, the rate of the change in the concentrations of camphene and isobornyl acetate gradually decreased with the prolongation of the reaction time. When the rate of change was zero, the reaction reached equilibrium. The reaction could quickly reach equilibrium by increasing the temperature, but when the temperature was above 90 °C, the reverse reaction and side reaction rates would increase. In this case, if the reaction time was prolonged, the GC content of isobornyl acetate would decrease. For example, the GC content of isobornyl acetate reached 80.5% after being held at 90 °C for 2 h. However, with the prolongation of the reaction time, the concentration change rate gradually became negative, and the GC content of isobornyl acetate in the final product also decreased.

#### 2.1.5. Effect of Water on Camphene Esterification Reaction

**Figure 6:**
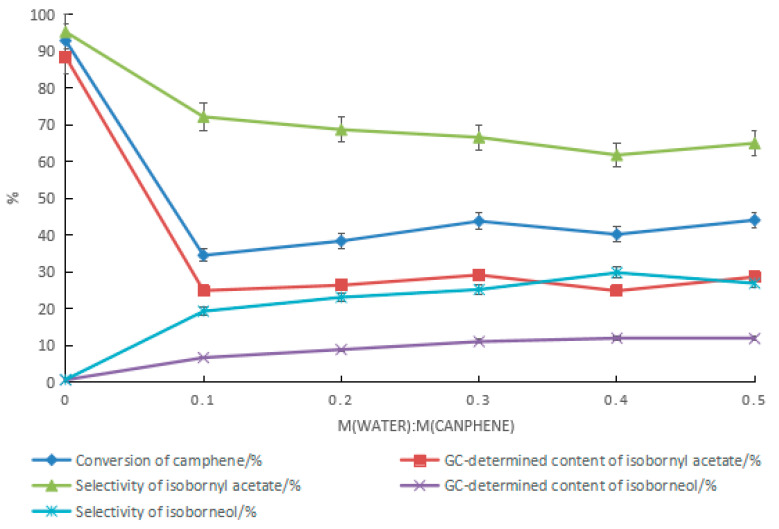
Effect of water on the reaction when tartaric acid–boric acid was used as the catalyst. Reaction conditions: m(camphene):m(acetic acid):m(tartaric acid):m(boric acid) = 10:25:0.5:0.4, reaction temperature of 70 °C, and reaction time of 18 h.

The effect of water on the synthesis of isobornyl acetate from camphene when tartaric acid–boric acid was used as the catalyst is shown in Figure 6. With the increase in the amount of water, the conversion rate of camphene and the GC content and selectivity of isobornyl acetate decreased, while the GC content and selectivity of isoborneol increased. Similar to previously reported results [18], when camphene reacted with acetic acid, adding water yielded a product containing isoborneol. In camphor synthesis factories, isobornyl acetate is mainly used for the synthesis of isoborneol, so isoborneol in the reaction product of camphene and acetic acid is beneficial. However, the presence of water reduces the conversion rate of camphene. As shown in Figure 6, the highest GC content of isoborneol in the product was 12.0%. The corresponding GC content of isobornyl acetate was 24.8%, and the conversion rate of camphene was 40.2%.

**Figure 7:**
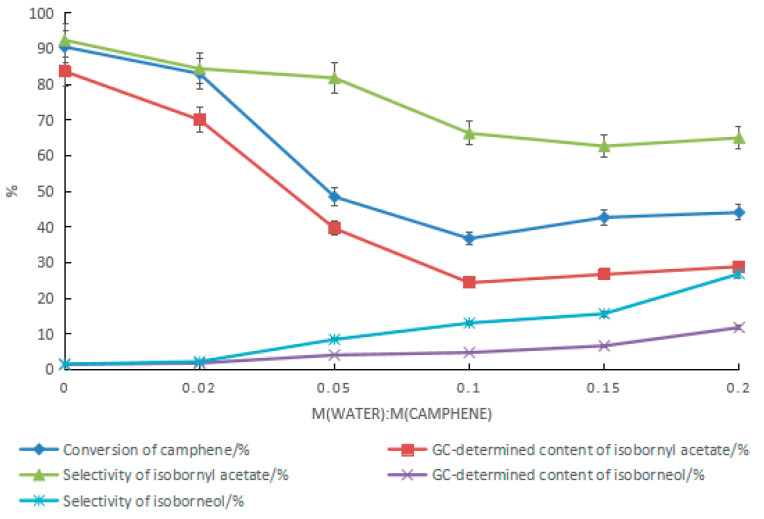
Effect of water on the reaction when mandelic acid–boric acid was used as the catalyst. Reaction conditions: m(camphene):m(acetic acid):m(mandelic acid):m(boric acid) = 10:25:1:0.4, reaction temperature of 70 °C, and reaction time of 18 h.

When mandelic acid–boric acid was used as the catalyst, the effect of water on the reaction was similar to that of the tartaric acid composite catalyst. As the amount of water increased, the conversion rate of camphene decreased rapidly. As shown in Figure 7, the maximum content of isoborneol was 11.8%. The corresponding GC content of isobornyl acetate was 28.9%, and the conversion rate of camphene was 44.1%. When camphene reacted with the acetic acid aqueous solution, there were two parallel reactions at the same time: camphene reacted with water to form isoborneol, and camphene reacted with acetic acid to produce isobornyl acetate. Isoborneol dehydrated easily to camphene, resulting in a low camphene conversion rate. Therefore, improving the content of isoborneol in the product is a meaningful and challenging task.

### 2.2. Reaction Mechanism and Product Analysis of the Esterification of Camphene Catalyzed by HCA–Boric Acid

**Figure 8:**
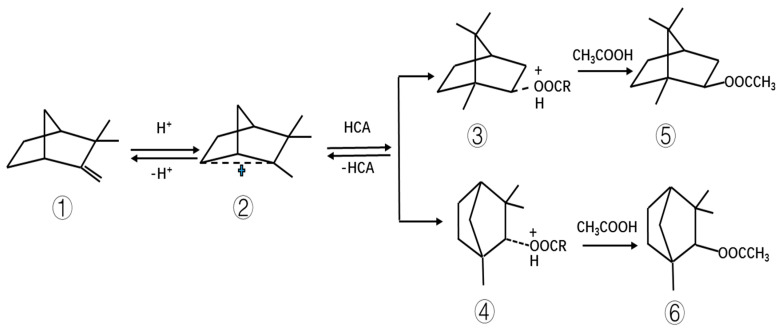
Mechanism of camphene reaction with acetic acid. Note: ① camphene, ② carbocation, ③ oxonium ion “isobornyl hydroxylate,” ④ oxonium ion “fenchyl hydroxylate,” ⑤ isobornyl acetate, ⑥ fenchyl acetate. R represents the alkyl group of HCA.

When the HCA–boric acid composite catalyst was used to catalyze the esterification of camphene, boric acid first reacted with the hydroxyl group of HCA to form a complex. In this reaction, the boric acid donated protons; these protons attacked the double bond of camphene, thus forming carbocation, as shown in Figure 8. After the rearrangement of carbocation, oxonium ions were formed with HCA, and then, under the attack of acetic acid, more stable isobornyl acetate and fenchyl acetate were formed.

Tartaric acid–boric acid was used as the catalyst to catalyze the reaction of camphene with acetic acid, and the product was analyzed via GC. The main components of the product were tricyclene, camphene, isoborneol, fenestrate acetate, and isoborneol acetate with GC contents of 1.2%, 5.9%, 0.6%, 2.4%, and 88.5%, respectively. Isobornyl acetate with a GC content of 98% was obtained through vacuum fractionation of the reaction product. The ^1^H-NMR spectrum of the synthesized isobornyl acetate is shown in the Supporting Information. The ^1^H-NMR results were as follows: (400 MHz, CDCl3) δ: 4.67 (t, J = 8.0 Hz, 1H), 2.02 (s, 3H), 1.82–1.65 (m, 5H), 1.61–1.53 (m, 1H), 1.32–0.98 (m, 6H), 0.84 (s, 3H), and 0.83 (s, 3H).

### 2.3. Catalyzed Synthesis of Isoborneol from Camphene

#### 2.3.1. Reaction of Camphene and Aqueous Acetic Acid

**Figure 9:**
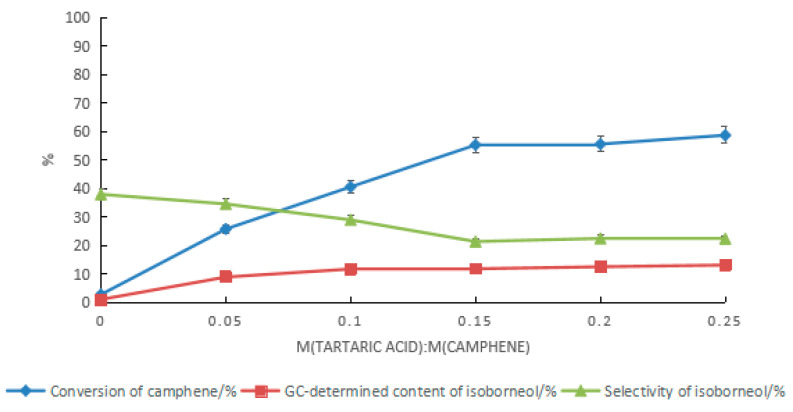
Effect of the tartaric acid dosage on the content of isoborneol in the product. Reaction conditions: m(camphene):m(acetic acid):m(water):m(boric acid) = 10:25:10:0.4, reaction temperature of 70 °C, and reaction time of 24 h.

To increase the conversion rate of camphene and the content of isoborneol in the product, with a mass ratio of water to acetic acid of 2:5, the conversion of camphene was promoted by increasing the catalyst dosage, as shown in Figure 9. With the increase in the tartaric acid content, the conversion rate of camphene increased, while the selectivity of isoborneol gradually decreased. In the product, the GC content of the isoborneol increased first and then became stable after the ratio of tartaric acid to camphene reached 0.15. When the ratio of tartaric acid to camphene was 0.25, the highest GC content of isoborneol in the product was 13.1%. At this time, the conversion rate of camphene was 58.7%, and the GC content ratio of isoborneol to isobornyl acetate was 1:3. Increasing the catalyst dosage was effective at promoting the conversion rate of camphene, but the effect of increasing the GC content of isoborneol in the product was limited. Even if the ratio of tartaric acid to camphene was increased to 0.45, the conversion rate of camphene was 72.3%, but the GC content of the isoborneol was 11.8%, and the ratio of isoborneol to isobornyl acetate in the product decreased to approximately 1:5.

**Figure 10:**
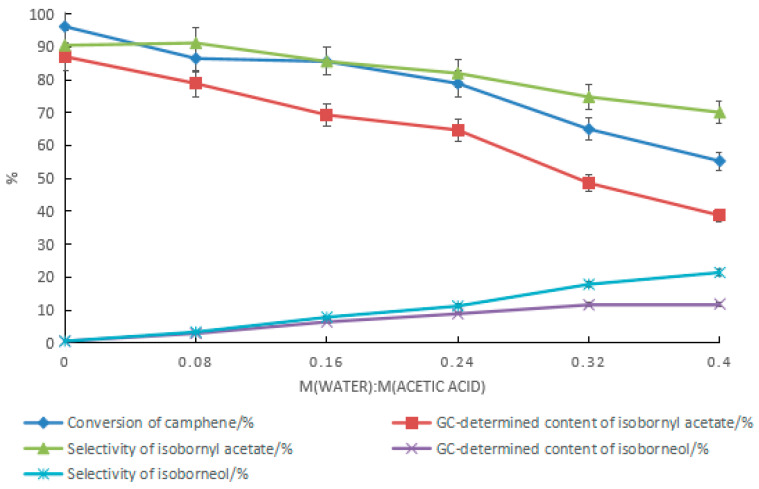
Effect of changes in the ratio of water to acetic acid on the product. Reaction conditions: m(camphene):m(acetic acid):m(tartaric acid):m(boric acid) = 10:25:1.5:0.4, reaction temperature of 70 °C, and reaction time of 24 h.

The ratio of water to acetic acid had a significant impact on the content of isoborneol in the product. As the ratio increased, the content and selectivity of isoborneol increased, as shown in Figure 10. During the synthesis of isobornyl acetate from camphene with tartaric acid–boric acid as the composite catalyst, when the ratio of water to acetic acid was 0.08, the conversion rate of camphene was 86.5%, and the GC contents of isoborneol and isobornyl acetate were 2.9% and 78.9%, respectively. However, with the increase in the ratio of water to acetic acid, the mass transfer resistance of the oil–water interface increased, and the conversion rate of camphene decreased rapidly. It can be seen that it was difficult to increase the GC content of isoborneol in the product only by increasing the ratio of water to acetic acid. The benefit of this is that for the synthesis of isobornyl acetate, acetic acid with a moisture content of no more than 7% can be used as a raw material, which is conducive to lowering material costs.

Further research showed that by increasing the ratio of water to acetic acid and adding ethyl acetate as a solvent, the GC content of isoborneol in the product could be improved. When the ratio of water, acetic acid, and ethyl acetate was 1:1:2, with the tartaric acid–boric acid composite catalyst, the GC content of isoborneol in the product reached 22.4%, the GC content of isobornyl acetate was 33.1%, and the GC content of the main by-product, fenchyl alcohol, was 6.6%. Under the above conditions, if titanium sulfate or zirconium sulfate was added, the GC content of isobornol in the product could reach 55.6%. This may have been because titanium sulfate and zirconium sulfate inhibited the generation of fenchyl alcohol and promoted the hydrolysis of isobornyl acetate, thereby increasing the content of isoborneol. Titanium and zirconium atoms have strong affinities for oxygen and hydrogen. In the composite catalytic system composed of titanium and zirconium atoms with HCA and boric acid, there may have been an electronic regulation mechanism similar to that reported previously. Their catalytic reaction mechanism requires further study.

#### 2.3.2. Camphene Hydration Reaction under Non-Solvent Conditions

Due to the large mass transfer resistance at the oil–water interface, a large amount of a hydrophilic organic solvent, such as isopropanol or acetone, needed to be added in the camphene hydration reaction. However, this introduces the problem that a large amount of solvent needs to be recycled, and at the same time, by-products such as isobornyl isopropyl ether and fenchyl alcohol also increase the difficulty of product separation and refinement [16]. If the camphene hydration reaction could be performed without a solvent, it would be conducive to the purification of the product and the reduction in the pollutant discharge.

Mandelic acid molecules contain hydrophilic carboxyl and hydroxyl groups as well as nonpolar phenyl groups, which are suitable for catalyzing the solvent-free camphene hydration reaction. Experiments showed that under the condition with no solvent, in the camphene hydration reaction when the mandelic acid–boric acid composite catalyst was used, the GC content of isoborneol in the product reached 26.1%, the selectivity of isoborneol was 55.9%, and the main by-products were camphene hydrate, tricyclic mandelic acid ester, and isobornyl mandeliate. The reaction formed intermediates of tricyclic mandelic acid ester and isobornyl mandeliate, and then isoborneol was obtained by the hydrolysis of the mandelic acid ester intermediates. By reducing the amount of water and increasing the dosage of mandelic acid, it was found that the main products became camphenate mandeliate and tricyclic mandelic acid ester, and the total GC content of mandelic acid ester was 57.9%. Then, the mandelic acid ester was separated, saponification was performed, and steam was applied to obtain isoborneol. The GC content of the obtained isoborneol reached 100%.

## 3. Experiment

### 3.1. Materials and Apparatus

The following starting materials and reagents were used in this study: camphene (≥75%), citric acid (99.5%), L (+)-tartaric acid (99.5%), DL-mandelic acid (99%), and boric acid (98%). These compounds were purchased from Macklin and Aladdin (Shanghai, China). Acetic acid (99.5%), ethyl acetate (99.8%), and sodium hydroxide (98%) were purchased from Chengdu Kelong Chemical (Chengdu, Sichuan Province, China). Distilled water was prepared in the laboratory.

The reaction apparatus was a PPV-3000 organic synthesis unit (EYELA, Tokyo, Japan). The following analytical instruments were used in this work: a 7890A gas chromatograph (Agilent, Santa Clara, CA, USA) equipped with quartz capillary chromatography columns (60 m × 0.25 mm × 0.25 μm) with AT-35 as the immobile phase, a TQ456 gas chromatography–mass spectrometry (GC-MS) instrument (Bruker, Billerica, MA, USA) equipped with BR-5 elastic quartz capillary columns (30 m × 0.25 mm × 0.25 μm) as chromatography columns, and an AVANCE III 400M nuclear magnetic resonance spectrometer (Bruker, Ferrandon, Switzerland).

### 3.2. Experimental Methods

Synthesis of isobornyl acetate: First, 10 g of α-camphene, 25 g of acetic acid, 0.1–1 g of tartaric acid or mandelic acid, and 0.1–0.5 g of boric acid were measured and added to a reaction bulb for magnetic stirring (500 rpm). The reaction temperature was controlled to 70 °C, and the reaction time was 16 h. After the reaction, the product was cooled and the catalyst was deposited at the bottom of the bottle. Through filtration, 90% of the catalyst could be recovered. The product was transferred to a separating funnel, and water was added to layer it. The upper layer was the product, and the lower layer was an acetic acid aqueous solution. The upper product was neutralized with alkaline water, washed, and dried with anhydrous sodium sulfate, after which sample analysis was performed. Isobornyl acetate was isolated by vacuum fractionation with a vacuum pressure of −0.085 MPa, and the fraction was collected at 120 °C.

Synthesis of isoborneol: First, 10 g of α-camphene, 10 g of acetic acid, 20 g of ethyl acetate, 1–5 g of water, and catalyst (4.5 g of tartaric acid/0.4 g of boric acid, or 0.8 g of titanium sulfate/0.4 g of citric acid) were measured and added to the reaction bulb for magnetic stirring (500 rpm). The reaction temperature was controlled at 70 °C, and the reaction time was 18 h. After the reaction was complete, the product was poured into the separating funnel for the standing and layering process. The upper layer was the product, and the lower layer was an acetic acid aqueous solution containing the catalyst. The upper products were neutralized with alkaline water, washed, and dried with anhydrous sodium sulfate, and then sample analysis was performed.

### 3.3. Analytical Methods

The proportions of the starting materials and products were calculated using the GC area normalization method.

For the GC analysis, high-purity nitrogen was used as the carrier gas, and the temperature program was as follows. The initial temperature was 70 °C (2 min of holding), with a first increase of 5 °C min^−1^ to 150 °C (3 min of holding), followed by a second increase of 10 °C min^−1^ to 230 °C (10 min of holding). The inlet temperature was set to 250 °C, and the total flow rate was set to 130.5 mL min^−1^, with a split ratio of 50:1 and a septum purge rate of 3 mL min^−1^. The analytes were detected using a flame ionization detector (FID), with a detection port temperature of 250 °C, a hydrogen flow rate of 40 mL min^−1^, an air flow rate of 450 mL min^−1^, and a nitrogen purge rate of 25 mL min^−1^. The injection volume was 0.2 µL.

For proton nuclear magnetic resonance (^1^H-NMR) data acquisition, the sample was placed into a sample measuring tube, CDCl_3_ was added, and the sample was analyzed by a 600-MHz nuclear magnetic resonance (NMR) instrument (frequency: 600.18 MHz), where the temperature was 296.9 K, the number of scans was 64, and the pulse width was 12.6 μs. The spectral width was 12,315.27, and the data point size was 32,768. The NMR spectra were processed by the Mestrenova software (Santiago de Compostela, Spain) and integrated after phase and baseline calibration.

## 4. Conclusions

The composite catalyst consisting of HCA and boric acid can catalyze the synthesis of isobornyl acetate from camphene. Under optimal conditions, the conversion rate of camphene and the GC content and selectivity of isobornyl acetate were 92.9.%, 88.5%, and 95.3%, respectively, when tartaric acid–boric acid was used as the catalyst, and they were 91.2%, 86.7%, and 95.1%, respectively, when mandelic acid–boric acid was used as the catalyst. The conditions under which the dosage of the tartaric acid composite catalyst was less than that of the mandelic acid composite catalyst were superior.

In the reaction of camphene and an acetic acid aqueous solution with HCA and boric acid as the catalyst, products containing isoborneol were obtained. In the synthesis of isobornyl acetate from camphene with tartaric acid–boric acid as the catalyst, when the ratio of water to acetic acid was 0.08, the conversion rate of camphene was 86.5%. The GC contents of isoborneol and isobornyl acetate were 2.9% and 78.9%, respectively. With the increase in the ratio of water to acetic acid, the GC content and selectivity of isoborneol in the product increased, while the conversion rate of camphene decreased rapidly.

By increasing the ratio of water to acetic acid and adding ethyl acetate as the solvent, the GC content of isoborneol in the product was increased. When the ratio of water, acetic acid, and ethyl acetate was 1:1:2 and the tartaric acid–boric acid composite catalyst was used, the GC content of isoborneol in the product reached 22.4%; when the titanium sulfate or zirconium sulfate–citric acid composite catalyst was used, the GC content of isoborneol in the product reached 55.6%.

Under conditions with no solvent, the mandelic acid–boric acid composite catalysts could catalyze the hydration reaction of camphene. The GC content of isoborneol in the product reached 26.1%, and the selectivity of isoborneol was 55.9%. By measuring the rate of change of the camphene or isobornyl acetate GC content in the product as the reaction time increased, we judged whether the reaction was at equilibrium. When the rate of concentration change was zero, the reaction had reached equilibrium. However, when the temperature was above 90 °C, as the reaction time increased, the rate of change in the concentration of isobornyl acetate was negative; this was caused by the increase in the reverse reaction and side reaction rates. The optimal reaction temperature of camphene esterification was 70 °C.

The composite catalyst composed of HCA and boric acid had much higher catalytic activity than the single-component catalyst and did not require a complicated preparation process. However, previous studies on the structure of the complex formed by boric acid and HCA mostly analyzed the results in aqueous solutions [21,22]. Although these studies provided us with a useful reference, the solubility of HCA and boric acid in camphene and acetic acid is very low, and thus, we could not completely reproduce the research results in aqueous solutions. Therefore, in different reaction systems, a considerable amount of work needs to be performed to fully understand the synergistic catalytic mechanism of HCA and boric acid. In particular, the ternary composite catalytic system composed of these compounds and metal elements could greatly improve the yield of camphene direct hydration reactions. The catalytic mechanism and reaction kinetics need to be studied in detail.

## Data Availability

All relevant data are included in the article.

## Supplementary Materials

The following supporting information can be downloaded at: https://www.mdpi.com/article/10.3390/molecules28041875/s1, Figure S1: Gas chromatography (GC) spectra of esterification products of camphene acetic acid catalyzed by tartaric acid–boric acid.; Figure S2: Proton nuclear magnetic resonance (^1^H-NMR) spectra of isobornyl acetate.; Figure S3: Product GC diagram of the reaction with ethyl acetate as the solvent and tartaric acid–boric acid as the catalyst. Reaction conditions: m(camphene):m(water):m(acetic acid):m(tartaric acid):m(boric acid):m(ethyl acetate) = 10:10:10:4.5:0.4:20, reaction temperature of 70 °C, and reaction time of 24 h; Figure S4: Product GC diagram of the reaction with ethyl acetate as the solvent and titanium sulfate–citric acid as the catalyst. Reaction conditions: m(camphene):m(water):m(acetic acid):m(titanium (IV) sulfate):m(citric acid):m(ethyl acetate) = 10:10:10:0.8:0.4:20, reaction temperature of 70 °C, and reaction time of 24 h; Figure S5: Product GC diagram of the solvent-free hydration reaction when mandelic acid–boric acid was used as the catalyst. Reaction conditions: m(camphene):m(water):m(mandelic acid):m(boric acid):m(ethyl acetate) = 13.6:3.6:6:0.18, reaction temperature of 70 °C, and reaction time of 24 h; Figure S6: Product GC diagram of the solvent-free hydration reaction when mandelic acid–boric acid was used as the catalyst. Reaction conditions: m(camphene):m(water):m(mandelic acid):m(boric acid):m(ethyl acetate) = 13.6:1.8:10.6:0.18, reaction temperature of 70 °C, and reaction time of 24 h; Figure S7: GC diagram of the steamed-out product after saponification reactions of camphenate mandeliate and isobornyl mandeliate. Note: 1. isoborneol; Formulas S1: conversion of camphene; Formulas S2: isobornyl acetate/isoborneol selectivity.
