# Supplementary material for: Study on Synthesizing Isobornyl Acetate/Isoborneol from Camphene Using α-Hydroxyl Carboxylic Acid Composite Catalyst
Source: Molecules. 2023 Feb 16;28(4):1875. doi: 10.3390/molecules28041875 (PMC9964953; doi:10.3390/molecules28041875)
Supplement: Supplementary file 1 [file molecules-28-01875-s001.zip › molecules-2183181-supplementary.pdf]

Supporting Information

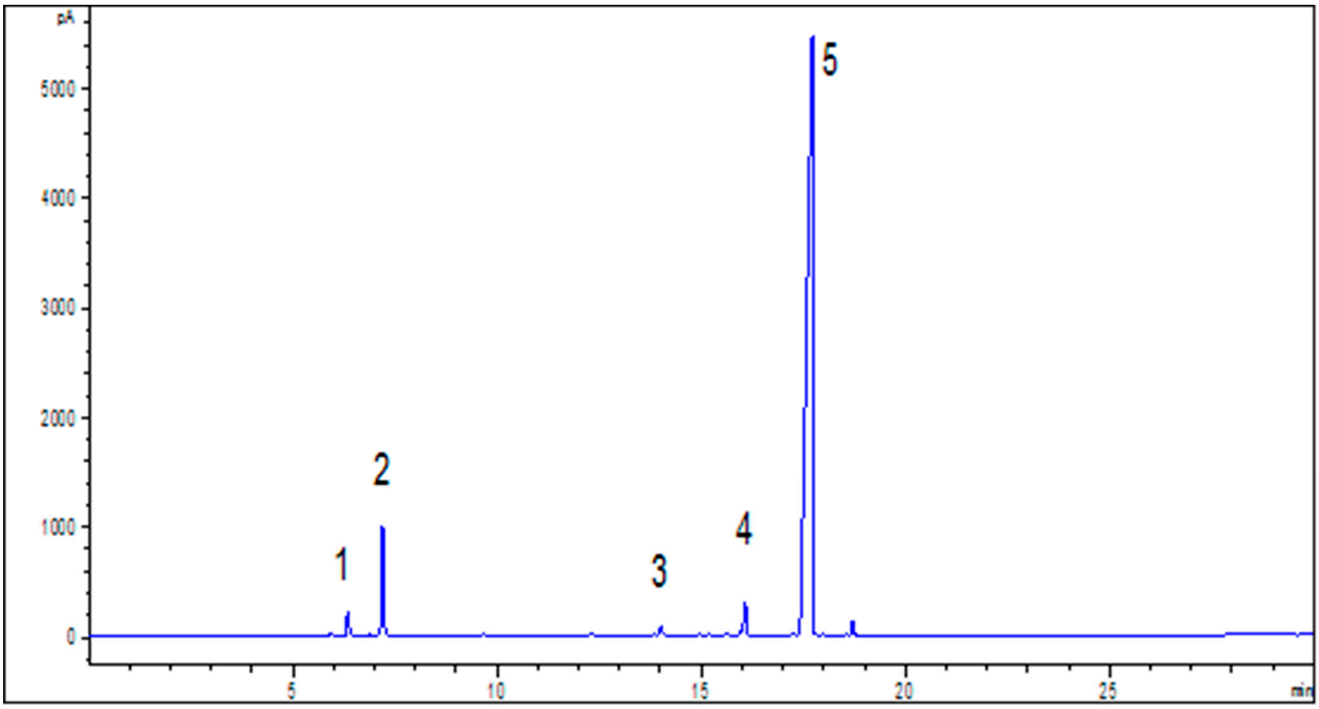

Fig. S1 Gas chromatography (GC) spectra of esterification products of camphene acetic acid catalyzed by tartaric acid–boric acid. Note: 1. tricyclene, 2. camphene, 3. isoborneol, 4. fenestrate acetate, and 5. isobornyl acetate.

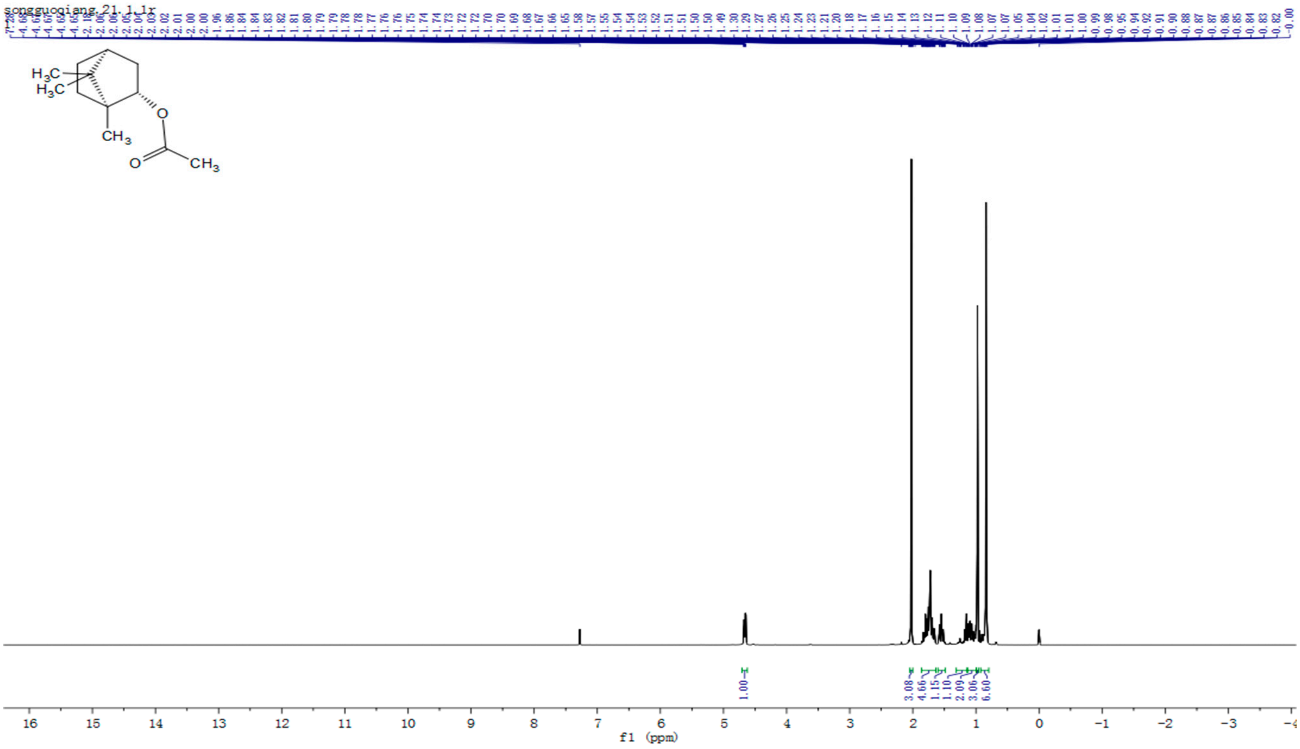

Fig. S2 Proton nuclear magnetic resonance (^{1}H-NMR) spectra of isobornyl acetate.

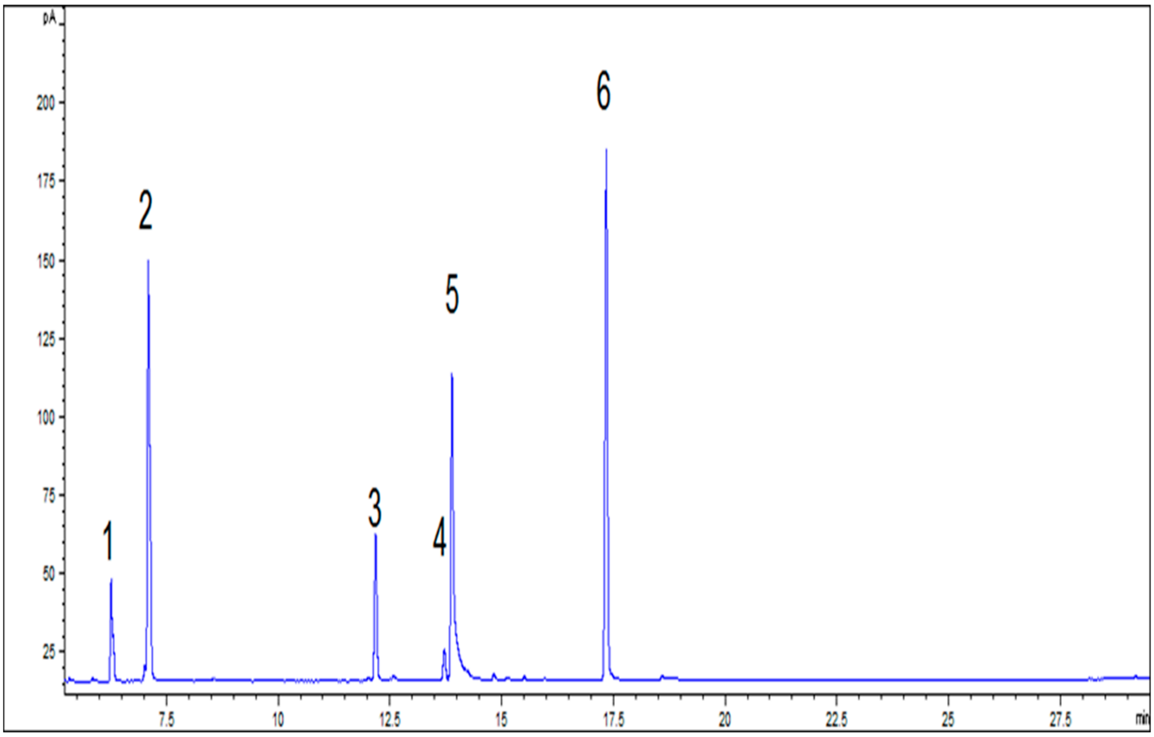

Fig. S3 Product GC diagram of the reaction with ethyl acetate as the solvent and tartaric acid–boric acid as the catalyst. Reaction conditions: m(camphene):m(water):m(acetic acid):m(tartaric acid):m(boric acid):m(ethyl acetate) = 10:10:10:4.5:0.4:20, reaction temperature of 70°C, and reaction time of 24 h. Note: 1. tricyclene, 2. camphene, 3. fenchyl alcohol, 4. camphene hydrate, 5. isoborneol, and 6. isobornyl acetate.

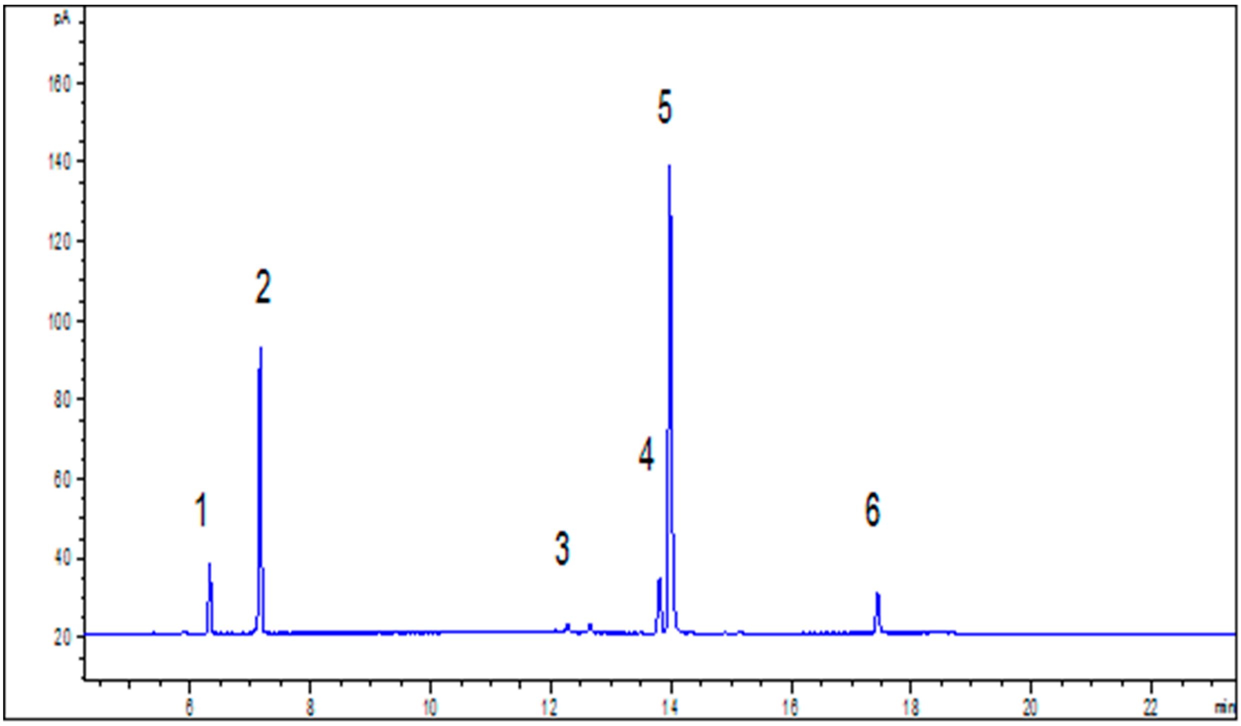

Fig. S4 Product GC diagram of the reaction with ethyl acetate as the solvent and titanium sulfate–citric acid as the catalyst. Reaction conditions: m(camphene):m(water):m(acetic acid):m(titanium (IV)

sulfate):m(citric acid):m(ethyl acetate) = 10:10:10:0.8:0.4:20, reaction temperature of 70°C, and reaction time of 24 h. Note: 1. tricyclene, 2. camphene, 3. fenchyl alcohol, 4. camphene hydrate, 5. isoborneol, and 6. isobornyl acetate.

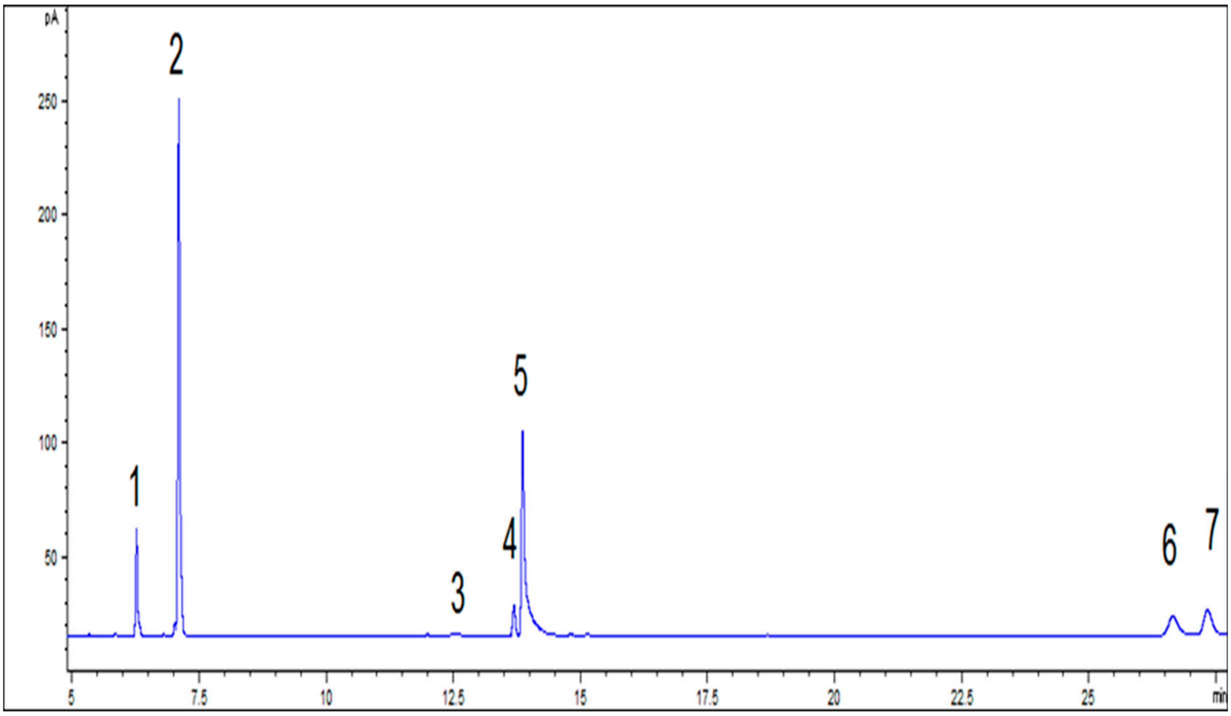

Fig. S5 Product GC diagram of the solvent-free hydration reaction when mandelic acid–boric acid was used as the catalyst. Reaction conditions: m(camphene):m(water):m(mandelic acid):m(boric acid):m(ethyl acetate) = 13.6:3.6:6:0.18, reaction temperature of 70°C, and reaction time of 24 h. Note: 1. tricyclene, 2. camphene, 3. fenchyl alcohol, 4. camphene hydrate, 5. isoborneol, 6. tricyclic mandelic acid ester, and 7. isobornyl mandeliate.

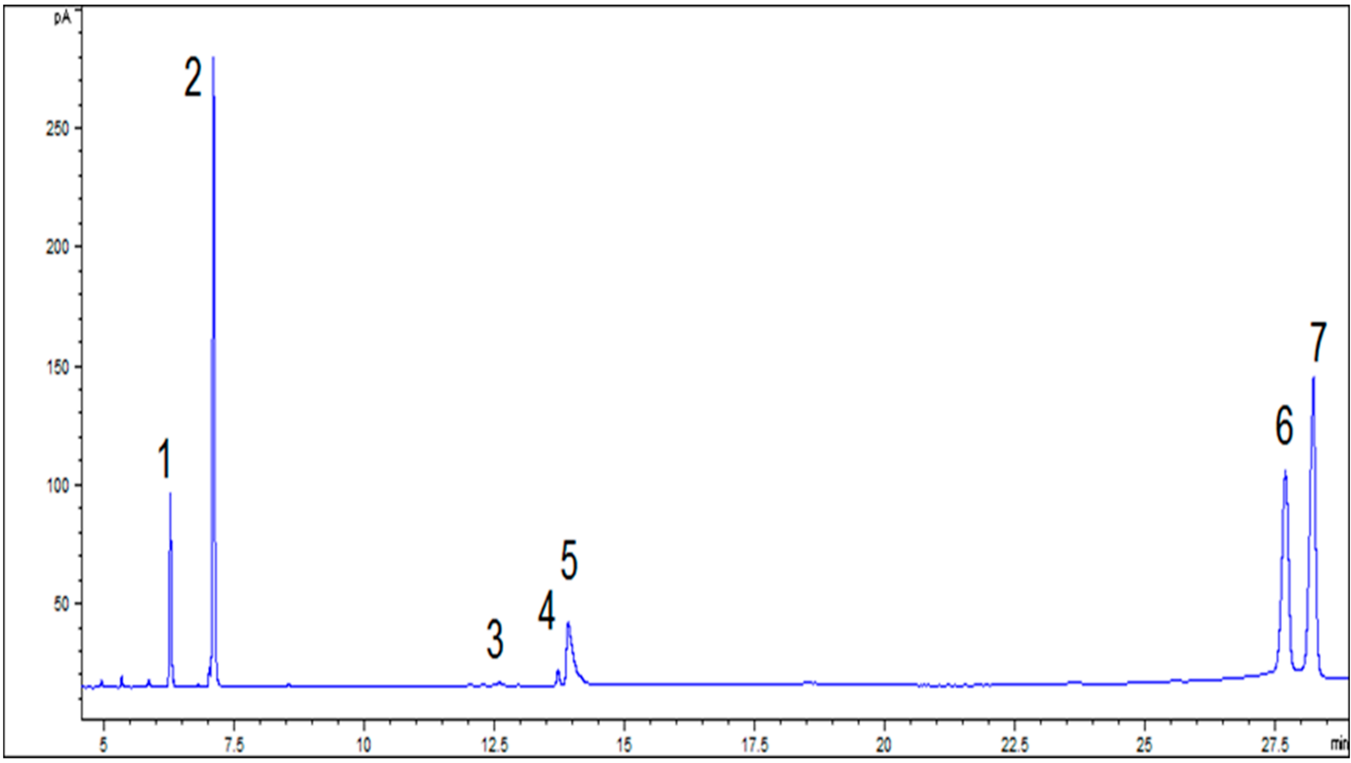

Fig. S6 Product GC diagram of the solvent-free hydration reaction when mandelic acid–boric acid was used as the catalyst. Reaction conditions: m(camphene):m(water):m(mandelic acid):m(boric acid):m(ethyl acetate) = 13.6:1.8:10.6:0.18, reaction temperature of 70°C, and reaction time of 24 h. Note: 1. tricyclene, 2. camphene, 3. fenchyl alcohol, 4. camphene hydrate, 5. isoborneol, 6. tricyclic mandelic acid ester, and 7 isobornyl mandeliate.

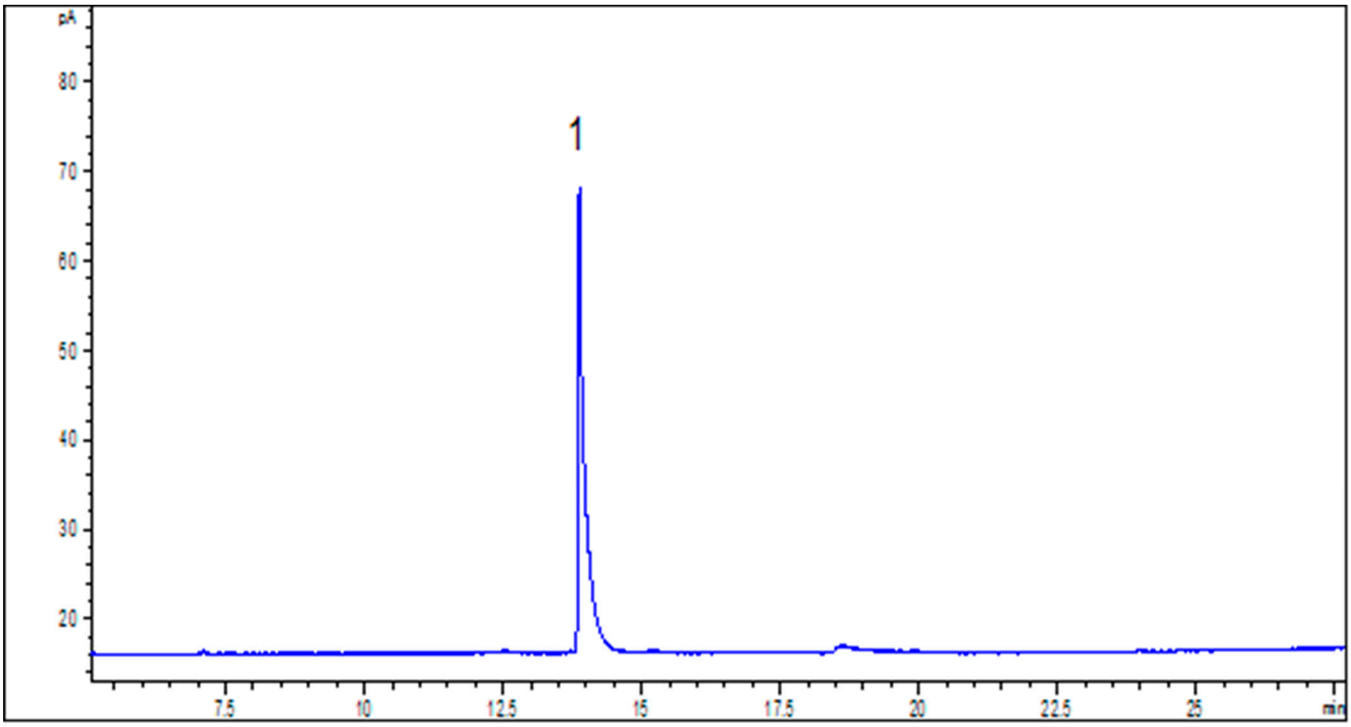

Fig. S7 GC diagram of the steamed-out product after saponification reactions of camphenate mandeliate and isobornyl mandeliate. Note: 1. isoborneol.

The conversion of camphene was estimated based on the following formula:

$$\text{Conversion of camphene} = \frac{\text{GC-determined content of camphene before reaction} - \text{GC-determined content of camphene after reaction}}{\text{GC-determined content of camphene before reaction}}, \quad (1)$$

The isobornyl acetate/isoborneol selectivity was estimated based on the following formula:

$$\text{Isobornyl acetate/isoborneol selectivity} = \frac{\text{GC-determined content of isobornyl acetate/isoborneol after reaction}}{\text{GC-determined content of camphene before reaction} - \text{GC-determined content of camphene after reaction}}. \quad (2)$$
